# Melanoma-derived Wnt5a conditions dendritic cells to promote regulatory T cell differentiation via the upregulation of indoleamine 2,3-dioxygenase: novel pharmacological strategies for augmenting immunotherapy efficacy

**DOI:** 10.1186/2051-1426-2-S3-P209

**Published:** 2014-11-06

**Authors:** Fei Zhao, Kathy Evans, Alisha Holtzhausen, Ciriana Orabona, Brent A Hanks

**Affiliations:** 1 Department of Medicine, Division of Medical Oncology, Duke University Medical Center, Durham, NC, USA; 2 Lineberger Comprehensive Cancer Center, University of North Carolina, Chapel Hill, NC, USA; 3 Department of Experimental Medicine, sect. Pharmacology, University of Perugia, Perugia, Italy

## 

**Figure 1:**
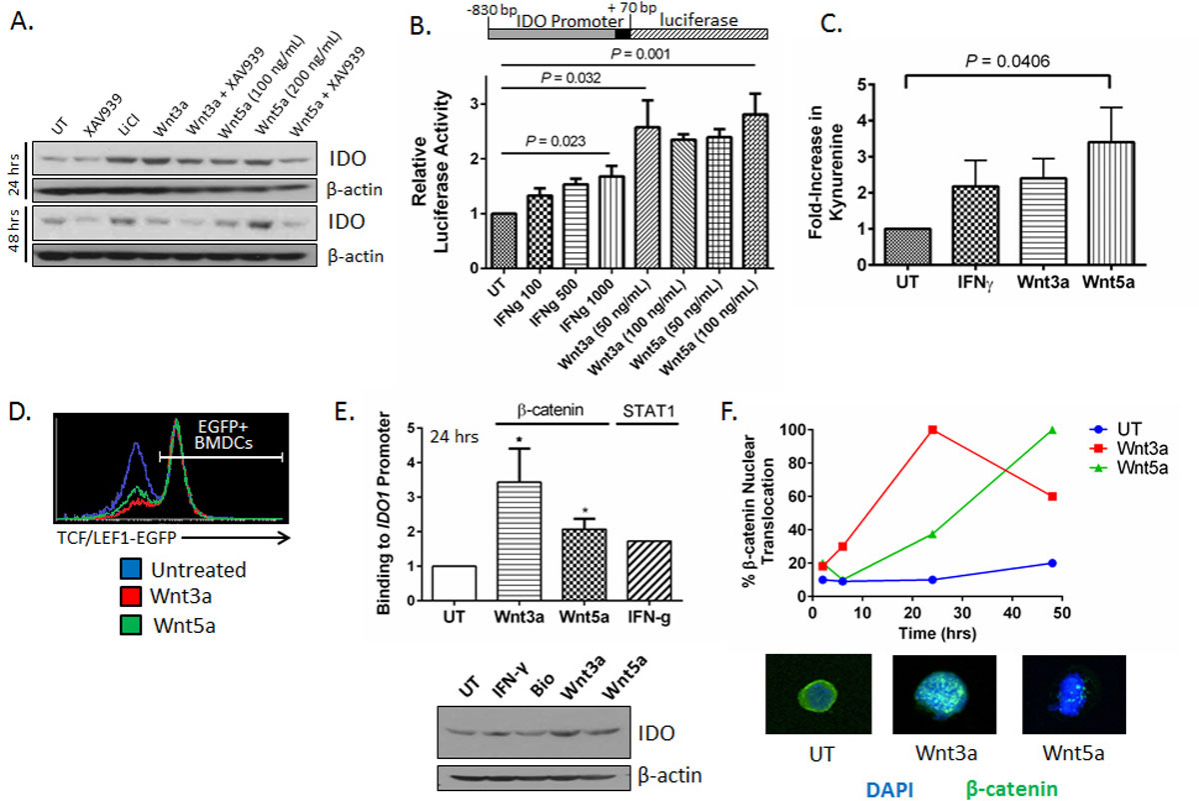
****Wnt3a and Wnt5a upregulate IDO expression and activity in DCs**. A**. Wnt5a induces durable IDO expression by BMDCs based on Western blot analysis at 24 and 48 hrs. Representative of 3 independent experiments. UT, untreated. XAV939, β-catenin inhibitor. **B**. Both Wnt3a and Wnt5a activate the IDO promoter in COS7 cells transiently transfected with a IDO_prom_-luciferase reporter plasmid. Performed in triplicate. Representative of 2 independent experiments. *P *value based on a one-way ANOVA. **C**. Wnt5a induces DC-derived IDO enzymatic activity based on kynurenine detection by HPLC. Cumulative data from 3 separate experiments. *P *value based on one-way ANOVA. **D**. Wnt3a and Wnt5a Stimulate β-catenin Activity in Primary TCF/Lef1-EGFP DCs *in vitro*. Representative of 2 independent experiments. **E**. Wnt3a and Wnt5a Induce β-catenin-IDO promoter binding in Primary DCs. Performed after 24 hrs of stimulation. IFN-γ induction of STAT1 binding to the IDO promoter serves as positive control. *Bottom*, Western blot generated from same samples. *P < 0.05. *P *value based on one-way ANOVA. **F**. β-catenin immunofluorescence microscopy confirms differential kinetics of Wnt3a- and Wnt5a-induced signaling in DCs. *Bottom*, examples of β-catenin nuclear translation in response to Wnt3a and Wnt5a (100x). 10 fields counted in triplicate. Representative of 2 independent experiments. All data is mean ± SEM.

**Figure 2:**
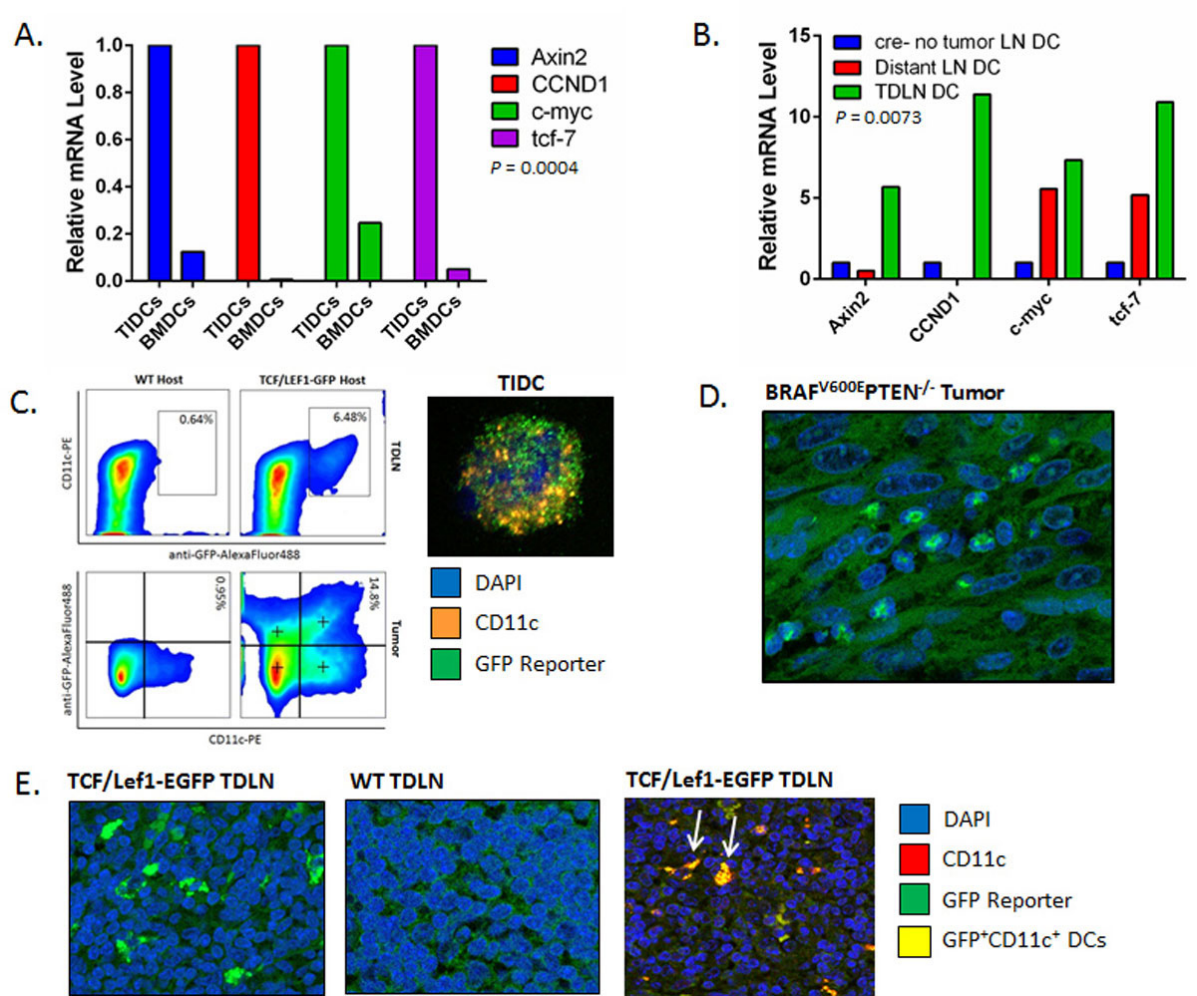
****Murine melanomas induce paracrine β-catenin signaling activation in tumor and TDLN DCs *in vivo***. A**.Tumor-infiltrating DCs (TIDCs) isolated from *Tyr::CreER;Braf^CA^;Pten^lox/lox ^*primary melanomas exhibit increased expression levels of β-catenin target genes, including *axin2, ccnd1, c-myc*, and *tcf-7*, based on qrt-PCR relative to BMDCs derived from non-tumor bearing mice. Representative of 2 independent experiments. *P *value based on a two-way ANOVA. **B**. DCs isolated from tumor-draining lymph node (TDLN) tissues harvested from melanoma-bearing *Tyr::CreER;Braf^CA^;Pten^lox/lox ^*mice exhibit elevated expression levels of β-catenin target genes relative to distant LN tissues harvested from the same mice as well as LN tissues harvested from non-tumor-bearing cre^- ^control mice. Representative of 2 independent experiments. *P *value based on a two-way ANOVA. **C**. β-catenin signaling activation in CD11c^+ ^DCs derived from primary BRAF^V600E^PTEN^-/- ^melanomas and TDLNs harvested from TCF/Lef1-EGFP reporter mice. *Left*, flow cytometry analysis and quantitation of GFP^+^CD11c^+ ^DCs. Representative of 2 independent experiments. *Right*, immunofluorescence confocal microscopy of a GFP^+^CD11c^+ ^DC isolated from a BRAF^V600E^PTEN^-/- ^primary melanoma. **D**. Confocal microscopy of a resected BRAF^V600E^PTEN^-/- ^melanoma resected from a TCF/Lef1-EGFP reporter mouse demonstrates a GFP-positive stromal infiltrate. Representative of 3 analyzed tumors. 60x. **E**. *Left*, Confocal immunofluorescence microscopy of TDLN tissue resected from TCF/Lef1-EGFP reporter mice demonstrates evidence of GFP^+ ^lymph node cells. *Center*, TDLN tissue resected from wild type (WT) BRAF^V600E^PTEN^-/- ^melanoma-bearing mice. *Right*, TDLN tissue from TCF/Lef1-EGFP reporter mice counterstained for CD11c expression shows evidence of GFP^+^CD11c^+ ^DCs (arrows). Representative of 3 analyzed lymph nodes. 60x.

**Figure 3:**
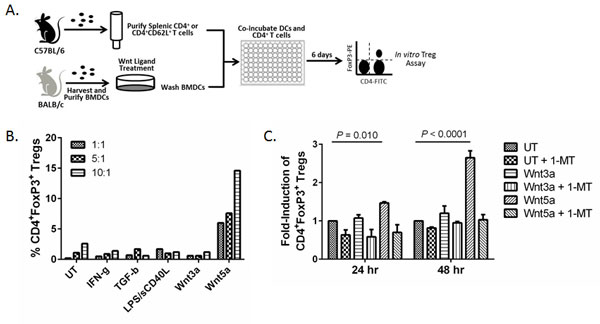
****Wnt5a conditions DCs to drive regulatory T-cell cifferentiation**. A**. Schematic of *in vitro *Treg assay. **B**. Wnt5a-conditioned DCs stimulate CD4^+^FoxP3^+ ^Treg differentiation *in vitro *following co-incubation with total splenic CD4^+ ^T cells at a 1:1, 5:1 or 10:1 T cell to DC ratio. CD4^+^FoxP3^+ ^Tregs were quantitated by flow cytometry. Representative of 3 independent experiments. **C**. DCs pre-conditioned for 24 and 48 hrs stimulate CD4^+^FoxP3^+ ^Treg differentiation *in vitro *following co-incubation with naive splenic CD4^+^CD62L^+ ^T cells. DCs were pretreated Wnt3a or Wnt5a for 24 or 28 hrs then co-incubated with splenic naïve CD4^+ ^T cells with or without the IDO inhibitor, 1-MT. CD4^+^FoxP3^+ ^Tregs were quantitated by flow cytometry. Performed in triplicate. Data is normalized to untreated DC condition. Representative of 3 independent experiments. *P *values based on a two-way ANOVA.

**Figure 4:**
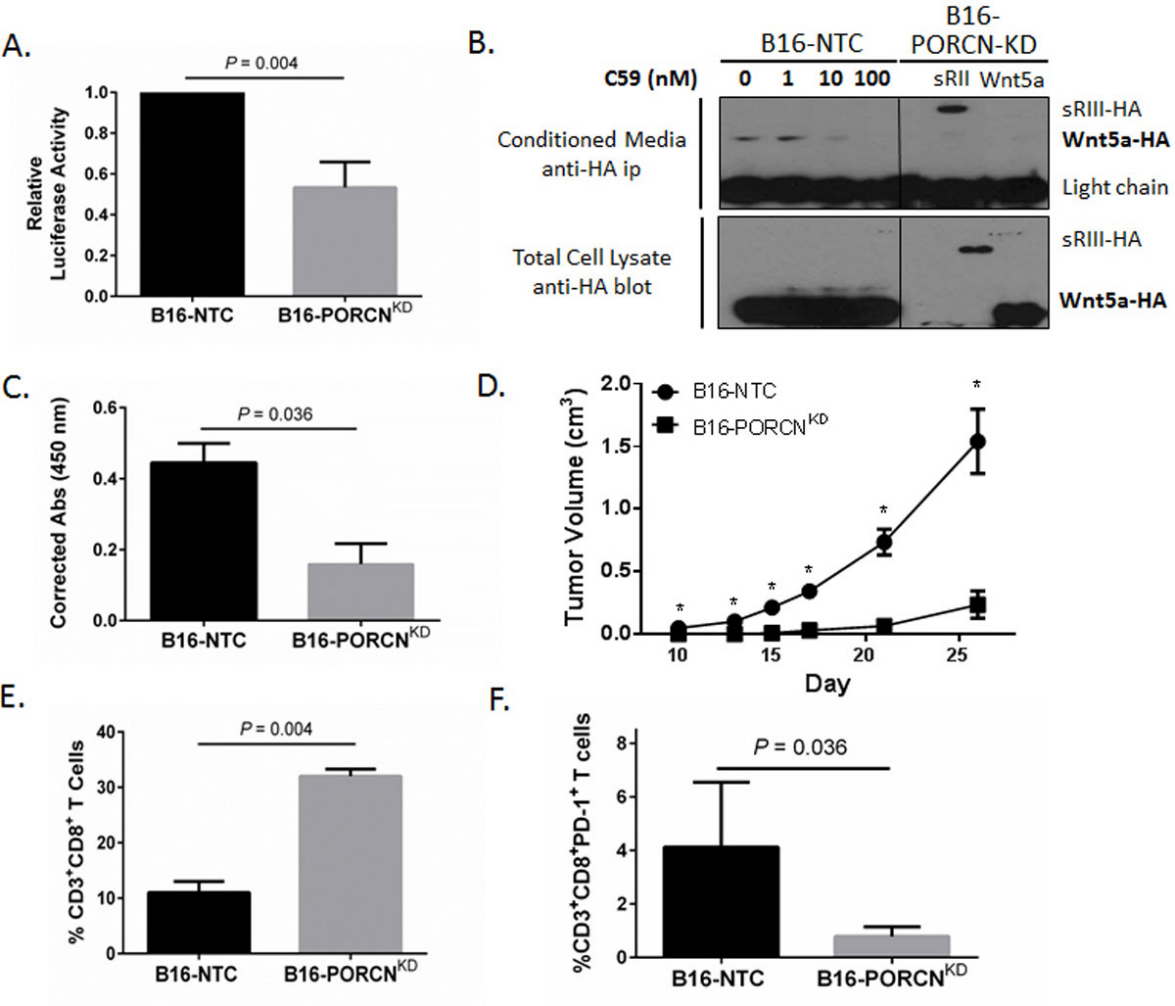
**Silencing PORCN expression in the B16 melanoma model enhances anti-tumor immunity and suppresses tumor progression *in vivo***. **A**. Silencing PORCN expression by B16/F10 cells suppresses paracrine β-catenin activation in a 293T-TCF/LEF1-luciferase reporter cell line. NTC, non-targeted control. KD, knock-down. Performed in triplicate. Representative of 3 independent experiments. *P *value based on two-tailed student's *t *test. **B**. PORCN silencing suppresses Wnt5a secretion from B16/F10 melanoma cells. *Top*, CM was harvested from HA-Wnt5a transfected B16-NTC cells treated with increasing concentrations of C59 (left) and HA-sRIII or HA-Wnt5a transfected B16-PORCN^KD ^cells (right) and immunoprecipitated with an anti-HA antibody. *Bottom*, Total cell lysates from HA-Wnt5a transfected B16-NTC were treated with increasing concentrations of C59 (left) and HA-sRIII or HA-Wnt5a transfected B16-PORCN^KD ^cells (right) were analyzed by anti-HA Western blot. HA-sRIII, HA-tagged soluble ectodomain of type III TGF-β receptor used as a positive control for protein secretion. Representative of two independent experiments. **C**. Wnt5a detection in the CM of B16-NTC and B16-PORCN^KD ^cells by ELISA. Cumulative data from 3 independent experiments. *P *value based on two-tailed student's *t *test. **D**. The B16-NTC and B16-PORCN^KD ^cell lines were implanted in C57BL/6 mice and tumor growth was monitored by orthogonal caliper measurements. 10 mice per group. Statistical significance based on Mann-Whitney U-test. *P < 0.05. **E**. Tumor-infiltrating CD3^+^CD8^+ ^T lymphocyte populations are increased in PORCN silenced B16/F10 tumors based on flow cytometry. Performed in triplicate. Representative of 2 independent experiments. *P *value based on two-tailed student's *t *test. **F**. Tumor-infiltrating CD8^+ ^T lymphocytes in PORCN silenced B16/F10 tumors exhibit diminished levels of surface PD-1 expression based on flow cytometry. Performed in triplicate. Representative of 2 independent experiments. *P *value based on two-tailed student's *t *test.

**Figure 5:**
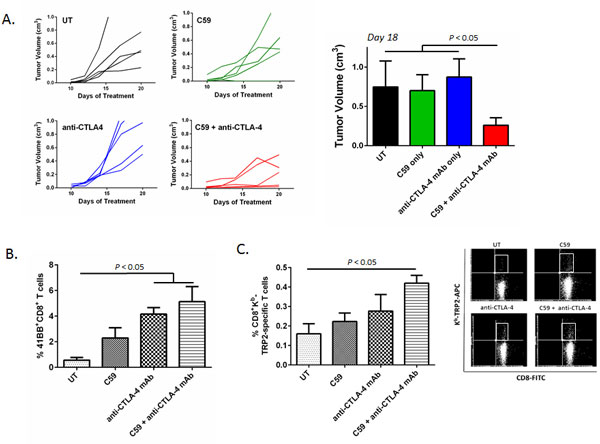
****C59 Inhibition of the Wnt-β-catenin signaling pathway synergistically enhances the efficacy of anti-CTLA-4 antibody immunotherapy in the B16 melanoma model**. A**. Combination inhibition of Wnt-mediated signaling and anti-CTLA-4 blockade synergistically suppresses B16 melanoma development *in vivo*. B16/F10 tumor-bearing mice were administered C59 alone at 5 mg/kg/day by oral gavage, anti-CTLA-4 mAb alone at 100 μg via intra-peritoneal injection every 3 days, or the combination. Control mice (UT, untreated) received daily vehicle control by oral gavage and isotype control antibody every 3 days. 5-6 mice per group. Representative of two independent experiments. **B**. Inhibition of Wnt secretion enhances the activation of B16/F10 melanoma-infiltrating CD8^+ ^T cells. Tumor-infiltrating 41BB^+^CD8^+ ^T cells in each treatment group were analyzed by flow cytometry. 4 mice per group. Representative of 2 independent experiments. *P *value based on a one-way ANOVA. **C**. Inhibition of Wnt secretion augments anti-CTLA-4 mAb-induced expansion of TRP2-specific CD8^+ ^T cells in B16/F10 melanomas based on dextramer flow cytometry analysis. 4 mice per group. Representative of 2 independent experiments. *P *value based on a one-way ANOVA.

Previous studies have shown the β-catenin signaling pathway to promote the development of tolerogenic dendritic cells (DCs) that are capable of driving regulatory T cell (Treg) differentiation. Interestingly, tolerogenic DCs have recently been described to play a role in carcinogenesis. However, the molecular mechanisms underlying the establishment of immune tolerance by this DC population are poorly understood and the methods by which developing cancers can co-opt this pathway to subvert immune surveillance are unknown. Using a genetically engineered model, we demonstrate that melanoma-derived Wnt5a ligand is a novel regulator of indoleamine 2,3-dioxygenase-1 (IDO) expression in local myeloid DCs and that Wnt5a induces the durable expression and enzymatic activity of IDO via β-catenin (Figures 1,2). Further, we show that Wnt5a-conditioned DCs promote Treg differentiation in an IDO-dependent manner and that melanoma secretion of Wnt5a both suppresses the generation of anti-tumor immunity and promotes melanoma progression *in vivo *(Figure 3). By genetically silencing the PORCN acyl transferase which is necessary for Wnt ligand secretion, we confirm the role of the soluble Wnt ligands in directing DC tolerization both *in vitro *and *in vivo *while also establishing a potential pharmacologic target for manipulating this novel pathway (Figure 4). Indeed, utilizing a small molecule inhibitor of PORCN, we are able to reverse Wnt5a-mediated IDO upregulation by DCs both *in vitro *and *in vivo*. These findings were expanded in further melanoma studies where we demonstrated small molecule PORCN inhibition to synergistically suppress melanoma progression while also enhancing anti-melanoma immunity in the setting of combination anti-CTLA-4 therapy (Figure 5). Additional work in human melanoma confirms the existence of this Wnt5a-mediated paracrine signaling pathway in DCs and reveals by microarray dataset analysis that human melanoma co-expression of Wnt5a and FoxP3 is highly significant. These data prompted us to hypothesize that a Wnt5a-induced gene signature in the melanoma microenvironment may be indicative of immune tolerance. Indeed, we have found that a Wnt5a gene signature identified in purified sentinel lymph node-derived DCs is associated with an inferior clinical prognosis in melanoma patients. This work emphasizes the importance of DC populations in directing tumor immune surveillance and illustrates that the molecular mechanisms involved in DC physiology represent potential targets for pharmacologically enhancing anti-tumor immunity.

